# The Role of the Microbiome in Connective-Tissue-Associated Interstitial Lung Disease and Pulmonary Vasculitis

**DOI:** 10.3390/biomedicines10123195

**Published:** 2022-12-09

**Authors:** Fotios Drakopanagiotakis, Elisavet Stavropoulou, Christina Tsigalou, Evangelia Nena, Paschalis Steiropoulos

**Affiliations:** 1 Department of Pulmonology, Medical School, Democritus University of Thrace, 69100 Alexandroupolis, Greece; 2 Service of Infectious Diseases, Department of Medicine, Lausanne University Hospital, University of Lausanne (Centre Hospitalier Universitaire Vaudois—CHUV), 1011 Lausanne, Switzerland; 3 Laboratory of Microbiology, Medical School, Democritus University of Thrace, 69100 Alexandroupolis, Greece; 4 Laboratory of Hygiene and Environmental Protection, Medical School, Democritus University of Thrace, 69100 Alexandroupolis, Greece

**Keywords:** microbiome, lung, connective-tissue diseases (CTD), interstitial lung diseases (ILDs), gut-lung axis, Sjögren’s syndrome

## Abstract

The microbiome can trigger and maintain immune-mediated diseases and is associated with the severity and prognosis of idiopathic pulmonary fibrosis, which is the prototype of interstitial lung diseases (ILDs). The latter can be a major cause of morbidity and mortality in patients with connective-tissue diseases (CTD). In the present review, we discuss the current evidence regarding microbiome in CTD-ILD and pulmonary vasculitis. In patients with rheumatoid arthritis (RA) the BAL microbiota is significantly less diverse and abundant, compared to healthy controls. These changes are associated with disease severity. In systemic sclerosis (SSc), gastrointestinal (GI)-dysbiosis is associated with ILD. Butyrate acid administration as a means of restoration of GI-microbiota has reduced the degree of lung fibrosis in animal models. Although related studies are scarce for SLE and Sjögren’s syndrome, studies of the gut, oral and ocular microbiome provide insights into the pathogenesis of these diseases. In ANCA-associated vasculitis, disease severity and relapses have been associated with disturbed nasal mucosa microbiota, with immunosuppressive treatment restoring the microbiome changes. The results of these studies suggest however no causal relation. More studies of the lung microbiome in CTD-ILDs are urgently needed, to provide a better understanding of the pathogenesis of these diseases.

## 1. Introduction

The human microbiome is defined as the whole microbes’ community, located in the cavities and on the surfaces of the human body [1]. The human microbiome consists of approximately ten to one hundred trillion bacterial, yeast, protozoa, and virus cells. Normally, these microorganisms co-exist with their human hosts in a symbiotic relationship. The microbiome plays a significant role in maintaining homeostasis and immune functions through cross-talk with the host immune system [1].

Connective tissue diseases and vasculitis are a heterogeneous group of disorders characterized by autoreactive, adaptive immune responses resulting in immune-mediated end-organ damage. The etiology of these diseases is not clearly defined. Genetic and environmental factors interplay to result in loss of immunological tolerance [2].

In the last century, significant changes have occurred in human environments and lifestyles. These changes are considered to have contributed to an imbalance of the symbiosis of the human host with the human microbiome, coinciding with a steep rise in immune-mediated diseases [3]. This imbalance refers to compositional and functional changes in the microbiome and is also described as ‘dysbiosis’ of the human host with their microbiome. The hypothesis that bacteria contribute to autoimmune phenomena is not new. Indeed, since the identification of Streptococcus pyogenes as the cause of ‘rheumatic fever’, numerous attempts have been made to define an etiologic relation between rheumatic diseases and microbes. However, such an etiologic association of one specific microbial agent with one specific autoimmune disease could not be established until now [2].

Interstitial lung diseases are a large group of pulmonary disorders, characterized histologically by the cardinal involvement of the pulmonary interstitium. These diseases manifest with dyspnea, cough, hypoxia, and impaired lung function. Many are associated with an underlying systemic condition, such as connective-tissue diseases, asbestos, and other dust exposure, and drugs or allergen exposure, such as hypersensitivity pneumonitis. However, in many cases the cause of interstitial lung disease is unknown. The prototype of the ILDs is idiopathic pulmonary fibrosis (IPF), an incurable disease, characterized by progressive distortion and loss of normal lung architecture through unchecked collagen deposition, resulting in traction bronchiectasis, reticulation, and honeycomb cyst formation. Recent evidence suggests that lung microbiome changes in patients with IPF are associated with worse prognosis and acute exacerbations of the disease [4]. Several studies have evaluated the lung microbiome in ILDs other than IPF, showing that the lung microbiome differs between ILDs [5,6,7].

Lungs can be variably affected in the setting of connective tissue diseases and vasculitis. The degree of pulmonary involvement can be mild, with only subtle radiographic changes, or progressive and severe, such as end-stage pulmonary fibrosis. Fulminant involvement, such as diffuse alveolar damage or diffuse alveolar hemorrhage can occur unexpectedly and be fatal if not early recognized and treated with aggressive immunosuppressive therapy. Indeed, interstitial lung diseases represent a major cause of morbidity and mortality in patients with connective tissue diseases and small-vessel vasculitis. Identification and treatment of interstitial lung diseases in the setting of autoimmune disorders are often challenging: a specific connective-tissue disease can have many different radiological and histological pulmonary manifestations. The opposite is also true: a pathology pattern can be found in a variety of connective tissue diseases [4]. Evidence suggests that the immune system or other disease-specific host factors may have a greater role in shaping the lung microbiome in CTD-ILDs [7].

The present review aims to describe current knowledge connecting the microbiome with CTD-ILDs and pulmonary vasculitis.

## 2. The Microbiome and the Lung

### 2.1. The Lung Microbiome

The lung is the largest human organ in direct contact with the environment. A total epithelial and airways area of 50–75 square meters is in constant exposure to ambient air, while the total volume of air that passes through the lungs is estimated to be 10,000 L/day. Despite this constant exposure of the lungs to the outside world, lungs have been traditionally considered sterile of bacteria. This was partly due to the failure of isolating bacteria in lung specimens with traditional culture techniques. The lung was considered non-sterile only in the setting of infections, such as pneumonia or bronchiectasis, as in such disorders microbes could be culture-isolated and considered pathogenic of the disease [6].

This notion has been questioned not only because microorganisms circulate in these 10,000 L of air that pass the lung, but also due to the knowledge that micro-aspiration of oropharyngeal and gastric content occurs in healthy individuals as well. The introduction of sequencing of the 16S rRNA gene technique allowed recognition of the fact that bacteria not only exist within the human lung, but they are altered in lung disease and correlate with alveolar immunity, and clinical outcomes [7,8].

Indeed, microbiome changes have been associated with ILDs, particularly its prototype, the IPF [6,9]. In stable IPF, a higher microbial burden is associated with a worse prognosis. In acute exacerbation of IPF higher microbial burden has been found in the lungs of patients, associated with higher morbidity and mortality. Although an association of lung microbiome with disease severity, the risk for disease progression and mortality has been established, causation has not yet been proven [6].

In hypersensitivity pneumonitis (HP), another common ILD, there is an increase in the *Staphylococcus* genus in the lung microbiome. Importantly, the lung microbiome differs between this disease and IPF [5]. Patients with HP exhibit a significantly lower lung bacterial burden compared with patients with IPF, although they still have a greater lung bacterial burden compared with healthy subjects. The bacterial burden is not associated with mortality in patients with HP, unlike IPF [5].

Other studies have shown lung microbiome changes in sarcoidosis and silicosis. In sarcoidosis, *Atopobium* spp. and *Fusobacterium* spp. were identified in the highest abundance compared to healthy subjects [10,11].

One common finding in ILD is the presence of traction bronchiectasis. Traction bronchiectasis is pathogenetically different from classical bronchiectasis related to inflammatory diseases, such as cystic fibrosis or recurrent childhood infections. The reason for bronchiectasis in ILD is the mechanical bronchus widening due to its traction from the shrinking, fibrotic lung parenchyma. Despite the different pathogenesis, traction bronchiectasis is also associated with reduced mucoid clearance and infection susceptibility [12]. Microbial populations develop often in areas of bronchial alteration. Modulator deficiency such as vitamin D deficiency plays a significant role in microbial population changes and is associated with more severe bronchiectasis and bacterial colonization [13]. Vitamin D deficiency has been reported in bronchiectasis-associated diseases, such as COPD and cystic fibrosis [14]. Interestingly, vitamin D deficiency is a common finding in ILDs, including CTD-ILD. In a study by Deng et al. vitamin D deficiency was a risk factor for CTD-ILD and serum calcium was a protective factor for CTD-ILD [15]. Similar findings have been reported in other ILDs, such as IPF [14].

Three factors seem to be involved in the composition of lung microbiome: (a) the instillation of microbiota originating from oral cavities, gastric content, and inhaled air, (b) the mucociliary clearance and cough reflex, which clear the microorganisms from the lung and (c) the local lung conditions, including oxygen partial pressure and temperature. A shift in the balance of these three factors as occurs in lung diseases can result in an altered microbiome [6].

The lung bacterial burden in healthy subjects is two-fold to four-fold lower compared to the oropharyngeal but of similar composition [16]. In addition, it is similar among individuals. The most prevalent phyla in the normal lung were reported to *be Bacteroidetes*, *Firmicutes*, *Proteobacteria* and *Actinobacteria*. The most prominent genera were *Prevotella*, *Streptococcus* and *Veillonella* [17,18,19]. Moreover, it seems that the lung microbiome—in contrast to the gastrointestinal tract microbiome- remains constant between various geographic regions [20].

### 2.2. The Gut Microbiome and the Gut-Lung Axis

The gut microbiome is defined as the diverse microbial communities that inhabit the host’s gastrointestinal (GI) tract [20]. The composition of the gut microbiome is stable within individuals and largely shared between healthy individuals. Normally, in the GI tract can be found up to 100 trillion microbes. The gut microbiome significantly affects human physiology and nutrition. Diet, age, antibiotics, lifestyle behaviors, and mode of delivery at birth are among the factors which contribute to the composition of the gut microbiome. Disruption of the gut microbiome is associated with various gastrointestinal and systemic diseases, including COPD, asthma and cystic fibrosis but also interstitial lung diseases (ILDs) [6,10,21].

At the phylum level, gut and lung microbiomes are similar and include *Bacteroidetes* and *Firmicutes*, however, there are differences at the species level. Since the gut and lungs are formed from the same embryonic tissue, their mucosal tissues have similarities in structure, and physiology [22]. Potential anatomic communications and complex pathways involving their respective microbiota have reinforced the existence of a gut–lung axis [22,23].

Gut microbiota alterations have been shown to modulate immune system responses in the lung by various mechanisms, including through regulatory T cells (Tregs), TLRs and inflammatory cytokines [24].

Emerging evidence suggests that dysbiosis in gut microbiota may contribute to the occurrence or development of several rheumatic diseases. In a recent systematic review, a decrease in a-diversity (i.e., the number of species in a sample) was found in rheumatic diseases. However, this finding was only seen in rheumatoid arthritis, systemic lupus erythematosus and polymyalgia while in systemic sclerosis an increase was observed. Another common finding in rheumatoid arthritis, Sjögren’s syndrome and systemic lupus erythematosus was the depletion of anti-inflammatory butyrate-producing microbe (i.e., *Faecalibacterium*) and the enrichment of pro-inflammatory microbe (i.e., *Streptococcus*) [25]. The role of gut microbiota in the prototype of ILDs, the IPF is not adequately described. Studies in animal models however suggest gut microbiota dysbiosis [26].

## 3. Rheumatoid Arthritis

Rheumatoid arthritis (RA) is a chronic disease that can cause irreversible joint damage and significant disability. It is characterized by synovial inflammation and joint erosion. Several mucosal surfaces have been implicated as sites of disease initiation in RA, including the periodontium, the lungs, the gut, and the genitourinary system. In animal models, major effects of oral as well as intestinal infectious triggers could be observed on the incidence and severity of arthritis. Studies trying to find potential causal associations between bacteria and RA, have identified *Porphyromonas gingivalis*, *Aggregatibacter actinomycetemcomitans*, and *Prevotella copri* as lead candidate pathogens [2].

Periodontitis, a chronic inflammatory disease of the gums has been associated with the pathogenesis of RA due to oral bacterial dysbiosis [27]. In a study of oral microbiota by patients with RA, subgingival microbiota in patients with new-onset RA was distinct from healthy controls [28]. *Porphyromonas gingivalis* in this study was directly associated with the periodontitis severity but not correlated with anti-citrulline peptide antibodies. Only *Prevotella* and *Leptotrichia* species were characteristic of new-onset RA irrespective of PD status and absent in controls [28]. Other studies have shown oral and gut dysbiosis in RA patients compared to healthy controls. No link can be made however between a specific pathogen and the development of RA [2].

Pleuropulmonary manifestations of RA are multiple and include interstitial pulmonary fibrosis, organizing pneumonia, lung nodules, bronchiolitis, bronchiectasis and pleural effusions [4].

Interstitial lung disease in RA has a male predominance. Smoking is a risk factor for the development of overt pulmonary fibrosis in patients with RA and is associated with the development of anti-citrulline-positive RA, in the presence of HLA-DR susceptibility genes. The MUC5B promoter variant gene has also been reported to be associated with the development of RA-associated ILD and specifically the usual interstitial pneumonia pathology pattern [4].

The lung represents a potential site of disease initiation in RA, and the finding of sputum IgA ACPA in RA and first-degree relatives may support this hypothesis [29]. Despite this, relatively little is known about the lung microbiome in RA [2].

Scher et al. used 16s RNA gene high-throughput sequencing to compare the bacterial community composition of bronchoalveolar lavage fluid (BAL) in patients with early, treatment-naïve RA, patients with lung sarcoidosis, and healthy control subjects [30]. Samples were further assessed for the presence and levels of anti-citrullinated peptide antibodies in both BAL and serum. The BAL microbiota of RA patients was significantly less diverse and abundant when compared to healthy controls, but similar to sarcoidosis patients. The overall lung bacterial community structure in RA patients harbored 40% fewer operational taxonomy units (OTUs) than healthy individuals. Taxa such as *Paraprevotellaceae*, *Chryseobacterium*, and *Burkhordelia,* while commonly found in healthy BAL, were less frequently present in samples from RA. RA BAL samples had a significant decrease in the families *Burkholderiaceae*, *Actinomycetaceae*, and *Spirochaetaceae*: 68% of the healthy control BAL contained *Actinomycetaceae* and 36% had *Spirochaetaceae*, compared to 5 and 0% in RA BAL, respectively. Surprisingly, *Porphyromonas* was isolated only in 10% of the BAL of the RA patients in contrast to 40% of healthy individuals. The same applied to the genus *Treponema* (which like *Porphyromonas* is highly associated with periodontitis) since it was only isolated in healthy controls. The authors further correlated the microbiome in the BAL with disease activity. RA disease activity was positively correlated with *Micrococcus* and *Renibaterium* at the genus level. Great similarities were found with the BAL of sarcoidosis patients, a pathogenetically different disease.

Importantly, the presence of the genus *Prevotella* correlated with rheumatoid factor IgA and anticitrulline peptide antibodies in patients with RA. These data support experimental studies of arthritis induction in mice due to *Prevotella nigrecens* and *Prevotella* isolation in the oral mucosa of RA patients [30].

The lung microbiome might also serve as a differential diagnosis marker among connective-tissue diseases. In a recent study, the BAL of patients with RA was compared with that of patients with dermatomyositis, another autoimmune disease, and controls regarding the microbiome. In contrast to the results of Sher et al., the authors found there were more operational taxonomic units (OTUs) in the lung microbiota of both RA and DM compared to controls, although there was no significant difference in the number of OTUs between RA and DM. Lung function decline, as a marker of pulmonary involvement due to autoimmune diseases, might explain this difference. 31 microbial biomarkers were clearly distinguished among RA, DM and controls. Seventeen genera including *Prevotella*, *Granulicatella*, *Rothia*, *Haemophilus*, *Veillonella*, *Stenotrophomonas*, *Leptotrichia* and *Actinomyces* predominated in the RA group [31].

The nasal microbiome shows also differences between RA and other autoimmune diseases. Nasal swabs of patients with RA, granulomatosis with polyangiitis (GPA) and healthy controls were collected and compared using 16S rRNA amplicon sequencing. A trend toward reduced microbiome diversity was detected in GPA samples compared with healthy controls and the abundance of bacterial taxa and microbial richness was significantly decreased in GPA samples compared with RA samples. Results should however be interpreted with caution since all patients were on immunosuppressive treatment [32].

## 4. Systemic Sclerosis

Systemic sclerosis (SSc) is an immune-mediated rheumatic disease that is characterized by vasculopathy, fibrosis of the skin and internal organs, such as the gastrointestinal (GI) tract, the skin, and the lungs, and often progresses to ILD [4].

Pulmonary manifestations of SSc include ILD, organizing pneumonia, chest wall restriction, and isolated pulmonary vascular disease. Pulmonary involvement has emerged as the major cause of excess morbidity and mortality in SSc. Asymptomatic ILD can affect up to 90% of the patients with SSc, although it may be clinically relevant only in 25% of patients. However, ILD accounts for about 19% of deaths in patients with SSc. Class II major histocompatibility complex associations increase the risk of ILD in SSc. The relative risk is increased if the anti-DNA topoisomerase (Scl-70) antibody is present. This autoantibody might be responsible for driving the immune response. A wide variety of cytokines in the lung contributes to the inflammatory cascade. Most important among them are interleukin-8, tumor necrosis factor-a, macrophage inflammatory protein 1a and RANTES. Accumulation of connective tissue matrix cells and proteins, with driving factor the profibrotic transforming growth factor-b as well as epithelial damage lead to pulmonary fibrosis [4].

Scleroderma changes in body surfaces can significantly affect the normal microbiome. Microbe dysbiosis on the other hand might be a factor influencing SSc pathogenesis and severity [2].

Various studies have demonstrated distinct microbial community differences between patients with SSc and healthy controls, revealing revealed enrichments in certain genera with pathobiont species (*Fusobacterium*, *Lactobacillus*, *g-Proteobacteria*, *Prevotella* spp.) and depletions in certain genera with commensal *species* (*Bacteroides*, *Faecalibacterium*, and *Clostridium*) in patients with SSc [33,34,35].

Dysbiosis in the gut might be the result of altered intestinal motility, secondary to vascular disease, neuropathy, fibrosis of the intestinal wall, and atrophy of intestinal smooth muscle.

In patients with SSc patients and severe GI involvement, *Prevotella* spp. have been mainly detected [33,34] and a decrease in *Clostridium* populations has been observed. *Clostridium* has been shown to determine the expansion of Treg cells, while *Prevotella* induces an immune response mediated by Th17 cells [33]; this imbalance might explain the transition to a pro-inflammatory state.

Until now, a few studies have examined the association of gut microbiome and SSs-ILD. We could not find any studies regarding the lung microbiome and its correlation to SSc-ILD.

A hypothesis regarding the pathogenesis of ILD in SSc might be that the upper GI microbiota is aspirated in the lung due to esophageal dysmotility, inducing inflammatory or fibrotic responses. Another mechanism might be that GI microbial input (e.g., priming of immune cells trafficking through the gut that return to circulation) may modulate fibrotic responses in distant organs, such as the lungs [36].

Andreasson et al. examined whether observed microbiome gut alterations in SSc are due to the disease itself or external factors, such as immunosuppression use or geographic region and whether microbiome alterations might be correlated with specific disease features, such as ILD. Two cohorts of SSc patients were examined, one in Sweden and one in the USA. The authors found that even in early SSc, microbial gut dysbiosis was present with an abundance of several genera deemed pathobiont (e.g., *Desulfovibrio*, *Ruminococcus*) and decreased abundance of several genera deemed commensal (e.g., *Faecalibacterium*). This finding suggests that GI microbes may contribute to early pathogenic processes in SSc. Another finding of the study was that the presence of ILD in SSc was independently associated with microbial composition. This result was consistent with previous findings of the same research group, showing that patients with SSc and ILD had increased dysbiosis scores compared with patients with SSc without ILD [36].

Calprotectin is a protein produced by activated macrophages and neutrophils. Fecal levels are increased when it aggregates due to neutrophilic inflammation. In patients with SSc, fecal calprotectin levels can identify patients with small intestinal bowel overgrowth, as an indirect marker of gut microbe dysbiosis. In a study that analyzed the link between gut inflammation and ILD in a large SSc population, increased fecal calprotectin levels strongly correlated with ILD progression. It could be assumed that increased fecal calprotectin is a biomarker of gut microbiome dysbiosis, which in turn affects the lungs through the gut-lung pathway. However, increased fecal calprotectin levels might simply depict increased GI-Involvement due to SSc and thus a generally more advanced disease [37].

Animal studies suggest the above-mentioned clinical observations. Mehta et al. reported the effect of GI-dysbiosis on the subsequent development of skin and pulmonary fibrosis in an experimental model of SSc [38]. Mice induced to have SSc-like disease received a single oral antibiotic dose (streptomycin) in early life. Streptomycin led to a shift in the gut microbiome towards a higher *Bacteroidetes*/*Firmicutes* ratio. Early life and continuous streptomycin administration was associated with progressive lung fibrosis compared to mice that were not given antibiotics, while the adult-life streptomycin administration did not lead to lung fibrosis progression. These are striking results, suggesting that events early in life, which are associated with microbiome dysbiosis, might be in adult life associated with autoimmune diseases [38].

Reconstitution of normal gut microbiome products as a treatment of SSc-ILD has been examined in animal models [39]. A reduction in butyrate-producing bacteria and an increase in proinflammatory bacteria have been observed in the intestinal microbiota of SSc patients. Butyrate is a short-chain fatty acid, produced by the fermentation of indigestible fiber by bacteria in the colon [39]. Butyrate is an energy source for intestinal epithelial cells and controls the development of microbial communities and differentiation of immune cells by mediating host-microbe interaction in the intestines [40]. It downregulates the release of proinflammatory cytokines from macrophages and dendritic cells (DCs) and facilitates the differentiation of regulatory T cells (Tregs). Butyrate might have a therapeutic effect in various animal models of rheumatoid arthritis, colitis, and obesity [40]. In an animal model of bleomycin-induced skin and lung fibrosis, butyrate ameliorated both the skin as well as the lung fibrosis. Butyrate restored the balance between alveolar and interstitial macrophages in the bleomycin-induced lung fibrosis model and it also influenced fecal microbiota composition, suggesting a potential role in the treatment of SSc [40].

## 5. Sjögren’s Syndrome

Sjögren’s syndrome is an autoimmune disorder characterized by lymphocyte infiltration of the lacrimal, salivary, conjunctival, and pharyngeal mucosal glands, with variable involvement of extra glandular tissue and autoantibody production (mostly directed against ribonucleoproteins TRIM21/Ro52/SS-A, Ro60, and La/SS-B. Lung involvement usually consists of lymphocytic infiltration similar to that seen in salivary glands and results in tracheobronchial disease or ILD. Clinically significant ILD in Sjögren’s syndrome may develop in 8–27% of patients and is associated with a poorer quality of life and increased mortality [4].

The result of the decreased secretion of salivary and lacrimal fluids seen in Sjögren’s syndrome, containing antimicrobial factors and the alterations of mucosal barriers increases the risk of developing dysbiosis [41].

Recent studies demonstrated that a lower *Firmicutes*/*Bacteroidetes* ratio in Sjögren’s syndrome patients was a main characteristic of the gut microbiota composition compared with healthy controls, indicating the existence of gut microbial dysbiosis in Sjögren’s syndrome [41].

However, the microbial composition of the buccal mucosa of patients with Sjögren’s syndrome did not differ significantly compared to non-Sjögren sicca patients and healthy controls. A higher *Firmicutes*/*Proteobacteria* ratio was observed in all groups. Oral microbiome changes were related to salivary secretion and disease activity. Between Sjögren’s syndrome patients and symptom controls, most of the studies observed no significant difference in the gut, oral, and ocular microbiota, which indicates that the changes in the microbiome might be only associated with mucosa dryness [41].

Other studies have shown contradictory results. A comparison between the oral microbiome and the clinical, laboratory, and radiological features of patients with Sjögren’s syndrome revealed the potential role of the salivary abundance of Lactobacillus and Streptococcus as biomarkers to differentiate Sjögren’s from non-Sjögren sicca patients [42].

In summary, data suggest that the severity of ocular and systemic features of Sjögren’s syndrome may associate with intestinal dysbiosis. The alterations of physiological mucosal barrier function in Sjögren’s syndrome may favor colonization with pathobionts or commensals that may elicit immune responses [2].

Specific studies regarding the role of the microbiome in the development of lung disease in Sjögren’s syndrome are lacking.

## 6. Systemic Lupus Erythematosus

Systemic lupus erythematosus (SLE) is a systemic autoimmune disease caused by a complex interplay of genes, hormones, and environmental factors. Despite recent improvements in understanding the etiopathogenesis of the disease, the pathogenesis of SLE remains unclear.

Pleuropulmonary manifestations are common in SLE, with 50–70% of SLE patients experiencing pleuropulmonary complications at some point in their disease course. Pulmonary manifestations include ILD, organizing pneumonia, diffuse alveolar damage, diffuse alveolar hemorrhage, pulmonary hypertension, shrinking lung syndrome, antiphospholipid antibody syndrome and pleural disease.

Significant disease resembling interstitial pulmonary fibrosis develops during follow-up in less than 5% of the patients [4].

Numerous studies have confirmed the presence of gut dysbiosis and the alteration of specific microorganisms in SLE [43]. The genera significantly more represented in SLE are *Rhodococcus*, *Eggerthella*, *Klebsiella*, *Prevotella*, *Eubacterium*, *Flavonifractor*, and *Incertae sedis*; conversely, the genera *Dialister* and *Pseudobutyrivibrio* are significantly reduced [44].

A meta-analysis of studies comparing the fecal microbiome of SLE patients and healthy controls showed a lower diversity in SLE patients with a lower abundance of *Ruminococcaceae* [45].

An enrichment of *Proteobacteria* (particularly *Proteobacterium copri*) as well as a decrease in *Firmicutes* has also been described. However, various families of *Firmicutes* (*Lactobacilli*, *Clostridicae* and *Lachnospirae*) have greater abundance in SLE mouse models [43,46,47].

*Proteobacteria* have been reported to be associated with intestinal inflammation [48], reflecting the inflammatory response in SLE patients. The altered *Firmicutes/Bacteroidetes* ratio seen in SLE seems to induce immune changes, similar to other autoimmune diseases and inflammatory bowel disease, in particular a shift to a Th17-mediated immune response [49].

Moreover, gut microbiome dysbiosis has been directly associated with the disease severity in SLE patients and with specific manifestations of the disease: the intestinal expansion of *Ruminococcus gnavus*, an obligate anaerobic species, correlates well to the overall disease activity and with anti-native DNA levels, prognostic for the development of lupus nephritis [50]. Intestinal barrier changes in SLE can lead to bacteria translocation to the lymph nodes, inducing autoimmune phenomena, a process described as ‘leaky gut’. Such observations have been made not only in animal models but in patients with SLE as well. The commensal bacterium *Enterococcus gallinarum* is such an example. Increased titers of *Enterococcus gallinarum* antibodies are associated with increased anti-ds DNA, anti-Sm, and anti-ribosomal P antibodies [1].

Autoimmunity in SLE should not be regarded however as a one-way pathway. SLE per se as well as immunosuppressive treatments affect the gut microbiome and increase the risk for bacterial infections and dysbiosis [3].

Attempts to reconstitute normal gut microbiome in SLE have shown promising results: fecal microbiome transplantation in patients with SLE was safe, could partially normalize gut microbiome and improved SLE symptoms [51].

No studies of the microbiome have been published in SLE-associated lung disease. It is theoretically possible however, that like SSc and RA-ILD, changes in the gut microbiome might affect the gut-lung axis and thus SLE activity in the lung. Such a hypothesis remains to be proved.

## 7. Dermatomyositis

Dermatomyositis (DM) is an idiopathic inflammatory myopathy that is characterized by the inflammation of the skeletal muscle and skin. ILD is considered a common systemic complication of DM. DM associated with ILD (DM-ILD) is one of the major extra-muscular manifestations contributing to increased morbidity and mortality [4].

Lou et al. have examined the lung microbiome of patients with dermatomyositis. They used 16S rRNA high-throughput sequencing to profile the bacterial community composition of BAL of patients with dermatomyositis associated with ILD. They found there were more operational taxonomic units (OTUs) in the lung microbiota in DM-ILD compared to healthy controls. The bacterial profile was similar but not the same as in patients with RA-ILD. Eight genera including *Corynebacterium*, 480_2_norank, *Aeromonas* and *Achromobacter* predominated in the DM-ILD. Taken together, these data showed that despite similarities between ILDs associated with autoimmune diseases, different microbiota can differentiate between the different ILDs [31].

Gut microbiome changes have also been reported in patients with DM [52]. Patients with DM trended toward lower microbial diversity compared with controls. At the phylum level, patients with DM had a significant shift to increased *Bacteroidetes* relative *to Firmicutes*, similar to results reported in patients with SLE. Microbial diversity is inversely correlated to disease severity in patients with DM. Patients with ILD had significant differences in microbial composition and lower microbial diversity compared with control subjects. In the ILD subgroup, a significant expansion of *Proteobacteria* was demonstrated. Proteobacteria is a known source of potent forms of bacterial LPS, a proinflammatory endotoxin that leads to endothelial damage, oxidative stress, and metabolic dysfunction. Indeed, *Proteobacteria* was not only expanded in the DM ILD but also positively correlated with genes involved in LPS biosynthesis and transport. The *Bacteroidetes* to *Firmicutes* ratio was also significantly higher in ILD patients compared to healthy controls and *Firmicutes* and *Actinobacteria* phyla were depleted in patients with ILD. Reduction of *Christensenellaceae* and *Ruminococcaceae* was reported in DM-ILD in the present study. Such a reduction has been associated with inflammatory diseases, metabolic dysfunction, and lipid metabolism [52].

## 8. Pulmonary Vasculitis

Pulmonary vasculitis is a clinicopathologic term encompassing a wide variety of individual disease entities, all of which share the presence of cellular infiltration within vessel walls, vessel destruction, and ultimately tissue necrosis within the lung. The lung is mainly affected in the setting of small-vessel vasculitis or anti-neutrophil cytoplasmic antibodies (ANCA)-associated vasculitis, i.e., in the setting of granulomatosis with polyangiitis (GPA), microscopic polyangiitis (MPA), and eosinophilic granulomatosis with polyangiitis (EGPA). Clinical manifestations of ANCA-associated vasculitis are the presence of nodules or cavities in the lungs, diffuse alveolar hemorrhage, pulmonary-renal syndrome, involvement of the upper airways and rarely interstitial lung disease [4].

Microbiome studies in ANCA-associated vasculitis have focused on the mucosa of the upper and lower respiratory tract. A microbial contribution to GPA has been assumed due to the destructive granulomatous inflammation that can affect the respiratory tract. The role of *Staphylococcus aureus* in the pathogenesis of GPA has been extensively investigated. *Staphylococcus aureus* colonizes the nasal mucosa of patients with GPA and detection of staphylococcal superantigen is increased in GPA and has been associated with an increased risk of disease relapse [53,54]. Moreover, antibiotic treatment with trimethoprim/sulfamethoxazole is effective in preventing relapses in localized disease, and this effect is thought to be mediated by reducing *Staphylococcus aureus* in the upper airways [55]. BAL culture identified *Staphylococcus aureus* in the lungs of over one-third of GPA patients compared to none of the concurrently studied IPF controls. Moreover, BAL supernatant from GPA patients acted as a growth factor for cultured *S. aureus*, while fluid from IPF patients and normal controls did not promote growth [56].

A pathogenic hypothesis is that microbes may be an important environmental activator of GPA. Cross-reactivity between host and bacterial peptides may induce pathogenic ANCA, which are associated with GPA. Low-grade infections may also evoke inflammatory cytokines which prime neutrophils for activation by ANCA or stimulate neutrophils to release neutrophil extracellular traps (NETs) embedded with ANCA antigens, further breaking immune tolerance and generating autoantibodies [57].

Microbiome studies have shown microbiome changes, searching for a role for *Staphylococcus aureus* in GPA. Wagner et al. investigated changes in the nasal microbiota including a detailed analysis of Staphylococcus spp. by shotgun metagenomics in patients with active and inactive GPA. Shotgun metagenomic sequence data were also used to identify protein-encoding genes. The authors found that patients with active GPA had a higher abundance of S. aureus, shown in culture and 16sRNA profiling, while healthy controls had a higher abundance of *S. epidermidis*. During long-term follow-up of patients with inactive GPA at baseline, a higher *S. aureus* abundance was not however associated with an increased relapse risk [58].

Lamprecht et al. compared the nasal microbiota in GPA and RA. They showed a reduced abundance of bacterial taxa and microbial richness in the GPA samples while *Staphylococcus aureus* colonization was significantly increased in the nasal microbiome of GPA compared to RA and healthy controls [32].

In another study, microbial DNA was isolated from nasal swabs of patients with GPA and healthy controls by 16S rRNA sequencing. Compared to controls, participants with GPA had a significantly different microbial composition, with a lower relative abundance of *Propionibacterium acnes* and *Staphylococcus epidermidis* [59]. *Staphylococcus aureus* was among the most abundant species identified, but no difference in the relative abundance of *Staphylococcus aureus* was observed between GPA and controls. *Malasseziales* was the most abundant fungal taxa in the nasal cavity. Disease activity in GPA was associated with a lower abundance of fungal order *Malasseziales* compared to participants with GPA in remission and controls. Interestingly, the use of non-glucocorticoid immunosuppression was associated with normalization of the nasal microbiome, with more dysbiosis and a lower abundance of *Propionibacterium acnes, Propionibacterium granulosum*, and *Staphylococcus epidermidis* primarily observed in GPA patients not on immunosuppressive therapy [59].

The same group has longitudinally searched microbiome changes due to disease activity and relevant nasal microbiome changes during relapses. Bacterial 16S gene sequencing was performed on nasal swabs of 19 patients with GPA followed for a total of 78 visits, including 9 patients who developed a relapse and 10 patients who remained in remission [57]. *Corynebacterium* and *Staphylococcus* were the most abundant bacterial genera across all nasal samples. Patients on remission maintained a stable ratio of *Corynebacterium* to *Staphylococcus* across visits. Surprisingly, patients who experienced a relapse showed a significantly lower ratio at the visit before relapse, followed by a higher ratio at the time of relapse. This change was associated with higher PR3-ANCA levels. Species-level analysis revealed associations between nasal *Corynebacterium tuberculostearicum*, which was abundantly found in the nasal mucosa of GPA and relapse in GPA, as well as the development of PR3-ANCA. This is an important finding since nasal *Corynebacterium tuberculostearicum* is considered to have a pathogenic role in chronic rhinosinusitis and to participate in the pathogenesis of asthma. Moreover, the presence of *Staphylococcus aureus* was independently associated with the abundance of *Corynebacterium tuberculostearicum*, a surrogate finding of previous studies, which have shown *Staphylococcus aureus-Corynebacterium tuberculostearicum* interactions [57].

The above-mentioned findings suggest the hypothesis that not just specific microbes are critical for the pathogenesis of ANCA vasculitis but rather the totality of the microbiome.

Most of the studies in GPA have explored the nasal microbiome. Gut dysbiosis data has also been published in preliminary form in patients with ANCA-associated vasculitis. An enrichment of *Dialister* and *Prevotella* taxa in active ANCA-associated vasculitis patients has been reported and a positive correlation between worsening gut dysbiosis and disease activity was also found [60].

## 9. Potential Microbiome Therapeutics for CTD-ILD and Vasculitis

Two strategies for the therapeutic modulation of the microbiome in CTD-ILDs and pulmonary vasculitis may have clinical interest: modulating the respiratory microbiota and modifying the gut microbiome in order to affect the gut-lung axis.

(i) Direct modulation of the respiratory microbiota with antibiotics and corticosteroids has shown benefit in the treatment of chronic lung diseases, such as bronchiectasis and COPD [61].

Regarding antibiotics, macrolides [62], doxycycline and trimethoprim-cotrimoxazole have been associated with better prognosis in animal studies of ILDs [6,7], by partially restoring the normal microbiome. Macrolides have been successfully used for the treatment of organizing pneumonia, a common pattern of ILD in CTD-ILDs [62]. As mentioned in the vasculitis section, trimethoprim-cotrimoxazole is effective in preventing relapses in localized disease of GPA, and this effect is thought to be mediated by reducing *Staphylococcus aureus* in the upper airways [55]. However, other studies have failed to show a relevant benefit of antibiotic use in ILDs: in a large clinical study in patients with IPF, trimethoprim-cotrimoxazole was not associated with better clinical or functional outcomes [63]. More focused approaches may be needed: D’Alessandro-Gabazza et al. showed that Staphylococcus nepalensis releases the pro-apoptotic peptide corisin, which induces alveolar cell apoptosis. Corisin is conserved in various Staphylococci and Staphylococci are in increased abundance in the lungs of patients with IPF [64]. Patients with IPF and other ILDs, including CTD-ILDs, suffer acute exacerbations of ILDs, which are dramatic, sudden, and often lethal deterioration events of unknown cause. It was shown that IPF patients with acute exacerbations have higher levels of corisin compared to IPF patients without exacerbations. Inhibition of the lung microbiome-derived pro-apoptotic peptide corisin could effectively reduce the acute exacerbations of pulmonary fibrosis in experimental models [64].

The use of corticosteroids in IPF, the prototype of ILDs has shown detrimental effects, particularly when combined with the immunosuppressant drug azathioprine and N-Acetylcysteine, maybe due to pulmonary microbiome changes [7]. We cannot extrapolate these results however for the treatment of CTD-ILDs and vasculitis, since immunosuppressive therapy is the standard of treatment for these diseases [65]. It is important to remember that the pathogenesis of CTD-ILDs and vasculitis greatly varies from the pathogenesis of IPF: while in CTD-ILDs a systemic inflammation is the hallmark of these diseases, IPF is a lung-restricted process, associated with minimal signs of lung inflammation [65]. The use of non-glucocorticoid immunosuppression is associated with the normalization of the nasal microbiome in GPA, with more dysbiosis observed in GPA patients not on immunosuppressive therapy [59]. Studies regarding the effect of immunosuppressive treatment on the pulmonary microbiome are currently missing.

(ii) Modification of the gut microbiome as a therapeutic means for ILDs is an intriguing idea [66]. Intestinal microbial metabolites are involved in the process of pulmonary fibrosis through immune regulation, energy metabolism, extracellular matrix accumulation and other pathways. Such metabolites include short-chain fatty acids, amino acids and bile acids [26]. Short-chain fatty acids (SCFA) are metabolized in the cecum and colon by the microbiota. Different microbes can produce different types and amounts of SCFAs. The main two SCFAs are butyrate acid and propionic acid. They can enter the circulation and can restore respiratory epithelial barrier dysfunction. In experimental models of lung fibrosis, butyrate acid pretreatment [67] and treatment could reduce the degree of pulmonary fibrosis as well as the degree of skin fibrosis, suggesting a possible therapeutic role in SSc [40]. Modulation of amino acids in the gut might also have a therapeutic role in pulmonary fibrosis: Glutamate acts pro-fibrotic by increasing collagen deposition from myofibroblasts and by making them apoptosis-resistant [68]. Arginine reduces collagen degradation and increases collagen deposition. Arginine depletion attenuates the proliferation, migration and invasion of fibroblasts, thus exerting an anti-fibrotic effect. Arginine however might also exert an anti-fibrotic action, by modulating immune disorders [26]. Bile acids have been shown to promote lung fibrosis in mouse models due to chronic microaspiration [69].

As described in previous sessions, gut microbe changes are seen in all autoimmune diseases and gut microbe restoration is associated with inflammation reduction [70,71,72]. Direct regulation of gut microbiota may be achieved with probiotics and fecal microbiota transplantation. Probiotics are defined as ‘live microorganisms, that when administered on adequate accounts confer a health benefit on the host’ [73]. Probiotics can induce an increase in SCFA, thus restoring the intestinal barrier and reducing inflammation. Probiotics have shown promising results in animal models of RA and SLE. Some probiotics, such as *Lactobacillus*, can restore dysbiosis and enhance intestinal barrier function and alleviate SLE symptoms. Data supports that probiotics may restore the abnormal T cell subsets, particularly the abnormal levels of naïve CD4+T, γδT, Tfh, Treg, and Th17 cells seen in SLE [74]. In a recent meta-analysis, probiotics reduced inflammation in patients with RA, measured with C-reactive protein levels [75]. Fecal microbiota transplantation (FMT) has been considered a very effective way to restoration of the normal gut microbiome in disease and has been tried in cases of resistant Clostridium difficile infections. However, obvious factors make such a treatment unattractive. In addition, its efficacy is not consistent and transmission of communicable diseases cannot be excluded [71]. An alternative approach might be an autologous FMT, with fecal samples stored before disease onset [71]. In patients with SLE FMT was feasible, safe and associated with symptom improvement [51]. In patients with SSc, FMT reduced lower gastrointestinal symptoms [76].

Vitamin D deficiency is frequent in CTD-ILDs [15], other ILDs such as IPF [14] and autoimmune diseases, such as SLE [70]. Vitamin D supplementation can restore the gut intestinal barrier integrity, by restoring gut microbiota [70] and protect from lung fibrosis in animal models [14].

Diet is a critical factor in regulating gut microbiota. Diet switching can lead to gut microbiota composition changes within 24 h. Higher pro-inflammatory dietary intake (animal-based diet with reduced fiber content) is associated with various respiratory infections, COPD and reduced lung function [77].

Drugs used for the treatment of CTDs and vasculitis can also affect the gut microbiota. Methotrexate, sulfasalazine, etanercept and tocilizumab for example have been shown to restore normal microbiota in patients with RA [78]. Pharmacomicrobiomic studies can help understand the role of the gut microbiome in the various efficacy of anti-rheumatic drugs in patients with autoimmune diseases [72].

Ideally, gut microbiota can be restored by specifically eliminating harmful members of the microbiome. Unfortunately, antibiotics and immunosuppressants are not specific enough to achieve this goal [71]. Bacteriophages might contribute to the destruction of pathobionts without affecting the host cells [71].

## 10. Future Implications and Limitations of Microbiome Studies in CTD-ILD and Pulmonary Vasculitis

In recent years, a significant increase in publications regarding the description of the microbiome in various diseases has been undertaken. Our thinking changes gradually from the concept of infection and bacterial colonization (a concept undoubtedly still useful) to the idea of an imbalance of various bacterial organisms, which normally reside in mucosal surfaces (microbiota) as a trigger for autoimmune, allergic and chronic inflammatory disorders [3]. This notion of microbiome dysbiosis is particularly relevant for the lung, which has been traditionally considered to be sterile of microorganisms.

**Figure 1:**
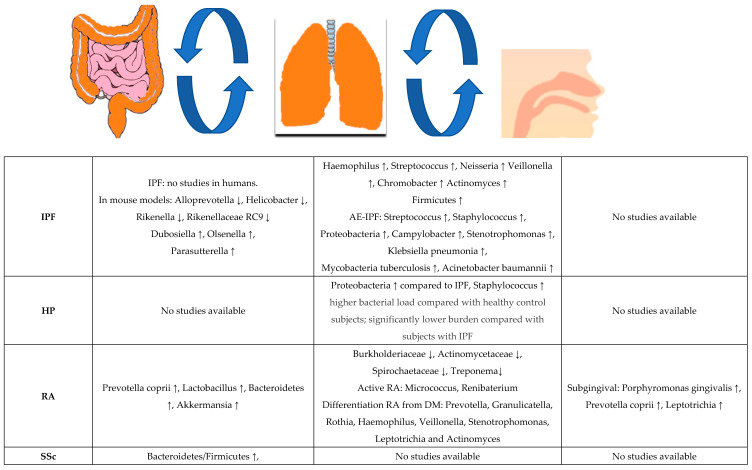

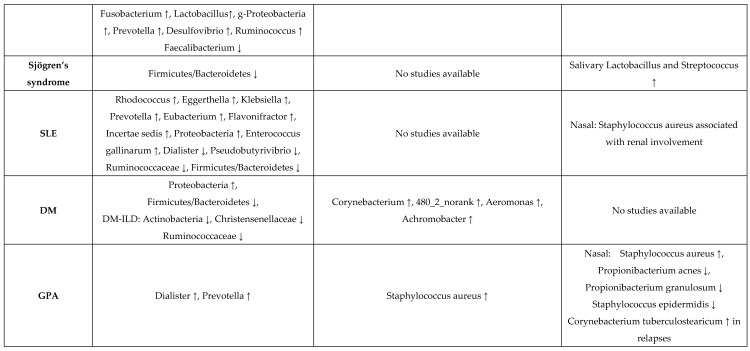
Microbiota changes in IPF, HP, CTD-ILDs and GPA. ↑: increased compared to healthy individuals, ↓: decreased compared to healthy individuals. Curved arrows depict the interactions between gastrointestinal tact and lung microbiota (gut-lung axis) and between lung and upper airways. For details see text.

Data regarding the association of microbiome and CTDs are accumulating; data regarding the association of CTD-ILDs with microbiome are still scarce. Particularly, the lung microbiome in these diseases has only begun to be described, in some diseases there are still no data available. Factors that might contribute to microbiome changes in CTD-ILD are summarized in Table 1. Microbiota changes in CTDs and vasculitis, as well as in the most common ILDs, IPF and HP are shown in Figure 1.

Several reasons partly explain the lack of lung microbiome studies in CTD-ILDs: in contrast to the gut, oral and nasal microbiome studies, for lung microbiome studies a bronchoscopy with BAL is required, a technique much more invasive compared to nasal and oral swabs or stool probes. In addition, although CTD-ILDs represent a significant cause of disability and mortality, in most patients with autoimmune diseases, the clinical course of the ILD is mild. ILD usually presents in patients with a long history of autoimmune disease which is often under immunosuppressive treatment, making it difficult for physicians to differentiate between interstitial lung disease as a manifestation of the underlying autoimmune condition or a side-effect of the treatment [4]. Moreover, therapeutic options for patients with CTD-ILD have increased in recent years, allowing the use of specific antifibrotic treatments such as nintedanib, for patients with progressive ILD [79]. However, the lung microbiome is not isolated but affected by the oral, nasal and gut microbiome (gut-lung axis) [57]. This particularly applies to diseases such as the SSc and dermatomyositis, characterized by decreased esophageal motility or the GPA vasculitis and Sjögren syndrome, showing similar histopathological changes in the upper and lower airways. Nasal, oral and GI-microbiome studies could still offer significant insights into the pathogenesis and serve as diagnostic and prognostic markers [3]. Challenges in research of lung microbiome in CTD-ILDs are presented in Table 2.

New studies are urgently needed in order to be able to identify the metabolic profile of CTDs and particularly of the ILD subtype.

## Figures and Tables

**Table 1:** Putative factors affecting the lung microbiome in CTD-ILDs and pulmonary vasculitis.

**Micro aspiration**
Esophageal dysmotility (SSc, dermatomyositis)
**Changes of the nasal and oral microbiome**
Nasal and oral ulcerations (GPA)
Xerostomia (Sjögren’s syndrome, primary and secondary, e.g., associated with SLE or RA)
**Changes of GI-Microbiome-Dysbiosis (gut-lung axis)**
Aberrant Th-17 responses and intestinal wall damage
Bacteria translocation and activation of the innate immunity due to secretion of pathogen-associated molecular patterns
Reduced production of anti-inflammatory short-chain fatty acids
**Immunosuppressive therapy**
**Impaired cough and mucociliary clearance**
Bronchiectasis (RA)
Extrinsic restriction of ventilation due to skin tightness of the chest wall (SSc)
Shrinking lung syndrome (SLE)
Xerotrachea, chronic bronchitis, small airway disease (Sjögren’s syndrome)
Respiratory muscle weakness (Myositis)

**Table 2:** Challenges in lung microbiome research in CTD-ILDs.

**Challenges in lung microbiome research in CTD-ILDs**
Invasive isolation techniques needed (BAL)
Radiologic interstitial changes often present, however in the minority of patients progressive
CTD-ILD usually clinically relevant years after first diagnosis of CTD
Patients under immunosuppressive treatment
Other CTD organ manifestations more prominent (GI tract, joints, skin)

## Data Availability

Not applicable.
